# Introgression of the Self-Pruning Gene into Dwarf Tomatoes to Obtain Salad-Type Determinate Growth Lines

**DOI:** 10.3390/plants13111522

**Published:** 2024-05-31

**Authors:** Lucas Medeiros Pereira, Gabriel Mascarenhas Maciel, Ana Carolina Silva Siquieroli, José Magno Queiroz Luz, Ana Luisa Alves Ribeiro, Camila Soares de Oliveira, Frederico Garcia Pinto, Brena Rodrigues Mota Ikehara

**Affiliations:** 1 Postgraduate Program in Agronomy, Institute of Agrarian Sciences, Federal University of Uberlândia, Uberlândia 38410-337, Brazil; lucas.medeiros.agro@ufu.br (L.M.P.); analuisaribeiro@ufu.br (A.L.A.R.); 2 Institute of Agrarian Sciences, Federal University of Uberlândia, Monte Carmelo 38500-000, Brazil; camila.soares@ufu.br; 3 Institute of Biotechnology, Federal University of Uberlândia, Monte Carmelo 38500-000, Brazil; carol@ufu.br; 4 Institute of Agrarian Sciences, Federal University of Uberlândia, Uberlândia 38410-337, Brazil; jmagno@ufu.br; 5 Institute of Exact Sciences, Federal University of Viçosa, Rio Paranaíba 38810-000, Brazil; frederico.pinto@gmail.com (F.G.P.); ikeharabe@gmail.com (B.R.M.I.)

**Keywords:** *Solanum lycopersicum* L., biotic stress, food, metabolomics, sustainable production

## Abstract

The use of dwarf plants in tomato breeding has provided several advantages. However, there are no identified dwarf plants (*dd*) containing the self-pruning habit (*spsp*). The aim of this work was to obtain future generations, characterize the germplasm, and select potential dwarf plants with a determinate growth habit to obtain Salad-type lines. The work was started by carrying out hybridization, followed by the first, second, and third backcrosses. Once F_2_BC_3_ seeds became available, the introgression of the self-pruning gene (*spsp*) into dwarf plants (*dd*) began. Three strains of normal architecture and a determinate growth habit were hybridized with two strains of dwarf size and an indeterminate growth habit, thus yielding four hybrids. Additionally, donor genotype UFU MC TOM1, the commercial cultivar Santa Clara, and the wild accession *Solanum pennellii* were used in the experiment. Agronomic traits, fruit quality, metabolomics, and acylsugars content were evaluated, and dwarf plants with a determinate growth habit were selected. Hybrid 3 exhibited the highest yields. Visual differences between determinate and indeterminate dwarf plant seedlings were observed. It is suggested to carry out five self-pollinations of the best dwarf plant determined and subsequent hybridization with homozygous lines of normal plant architecture and determinate growth habit to obtain hybrids.

## 1. Introduction

The tomato plant (*Solanum lycopersicum* L.) is one of the most widely grown and consumed vegetables in the world. Among the world’s top 10 tomato producers, China stands out with a production of 38.7 million tons, followed by the United States and India (12.7 and 12.4 million tons, respectively). Brazil occupies the ninth position in this ranking with 3.6 million tons, corresponding to an area of 51.9 thousand hectares [1].

Tomato production is directed toward industrial processing or in natura consumption, and tomatoes can be classified as Cherry, Santa Cruz, Italian, Saladette, and Salad [2,3]. Salad tomatoes are preferred for fresh consumption and are the main form sold in Brazil [2,4,5]. In terms of growth habits, tomato plants can be divided into determinate and indeterminate. The latter shows continuous growth and requires cultural treatments such as training, weeding, and apical pruning [6], whereas the former are more compact and have higher quality fruit. The difference in plant growth habits is controlled by the recessive self-pruning gene [7,8].

Determinate growth tomato plants are becoming more popular than their indeterminate counterparts around the world. This is primarily because these plants require less space to cultivate and can be harvested automatically, their fruits ripen uniformly and exhibit high nutritional value, and their cultural treatments are cost-effective [9,10].

In this context, dwarfism genes have been explored in several cultivated species to obtain more compact and productive plants: wheat [11], rice [12,13,14]), maize [15,16,17], sorghum [18], soybean [19], peach [20], barley [21], and coffee [22]. There are reports that plant height is controlled by the association of phytohormones [23,24]. Several mutants and genes responsible for governing the height in tomato plants have been reported: Mutants self-pruning (*sp*) [25], semideterminate (*sdt*) [26], and suppressor of sp (*ssp*) [27] affect the number of internodes, while mutants brachytic (*br*) [28], dwarf (*d*) [29], elongated internode (*EI*) [30], gibberellin deficient-1 (*gib-1*), *gib-2*, *gib-3* [31], procera (*pro*) [32], short internode (*si*) [33], tomato internode elongated-1 (*tie-1*) [34], and *SlGID1a* gene [24] affect internode length. In tomato, the use of dwarf plants is made possible through the use of the UFU MCTOM1 lineage, due to the fact that inheritance is monogenic and recessive, with 3:1 Mendelian segregation in the F2 generation [35,36,37].

There are research reports that demonstrate the potential of using dwarf plants but with indeterminate growth habits [38,39,40,41,42] limiting obtaining determinate hybrids. However, to date, there are no studies on obtaining and selecting dwarf plants with a determinate growth habit of the Salad-type. Thus, the aim of this work was to obtain advanced generations, characterize the germplasm, and select potential dwarf plants with a determinate growth habit to obtain Salad-type lines.

## 2. Results

### 2.1. Agronomic Variables

**Figure 1:**
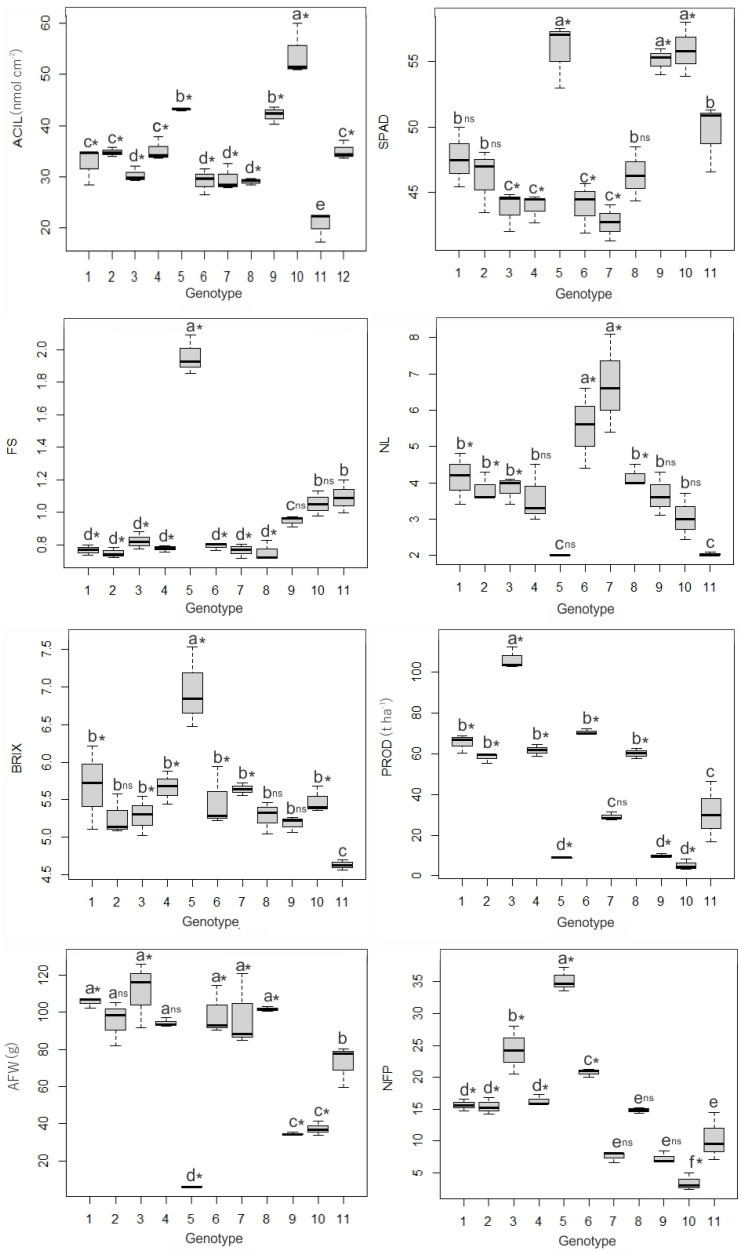
Mean values for agronomic traits: acylsugar content in nmol cm^−2^ of leaf area (ACIL), indirect chlorophyll content (SPAD), fruit shape (FS), number of locules (NL), soluble solids content (BRIX), yield in tons per hectare (PROD), average fruit weight in grams (AFW), and number of fruits per plant (NFP). 1. Hybrid 1 (UFU-057♀ × UFU-DTOM 441111♂); 2: Hybrid 2 (UFU-T7A♀ × UFU-DTOM 441111♂); 3: Hybrid 3 (UFU-T4R2#4♀ × UFU-DTOM 441111♂); 4: Hybrid 4 (UFU-T7A♀ × UFU-DTOM 19142121). 5. DP (UFU MC TOM 1 donor parent); 6: Female Parent 1 (UFU-057♀); 7: Female Parent 2 (UFU-T4R2#4♀); 8: Female Parent 1 (UFU-T7A♀); 9: Male Parent 2 (UFU-DTOM 441111♂); 10: Male Parent 3 (UFU-DTOM 19142121♂); 11: Santa Clara cultivar; 12: *Solanum pennellii* (LA-716). Averages followed by equal letters do not differ by Scott–Knott’s test at the 0.05 significance level. * significant and ^ns^ non-significant by Dunnett’s test at 0.05 significance compared to the Santa Clara cultivar.

Significant differences were observed for all the variables analyzed using the Scott–Knott test at a 0.05 significance level. The individual comparison of each treatment with the Santa Clara cultivar (Dunnett’s test, 0.05 significance level) also showed significant differences (Figure 1).

Dwarf plants exhibited the highest acylsugar contents, with the highest value obtained for male parent UFU-DTOM 19142121 (54.09 nmol cm^−2^), followed by male parent UFU-DTOM 441111 (42.10 nmol cm^−2^) and donor parent (43.16 nmol cm^−2^). Hybrids exhibited intermediate ACIL values similar to the wild access LA-716. The lowest value was observed in the commercial cultivar Santa Clara (20.72 nmol cm^−2^), which differed from the other treatments.

The SPAD index was highest in dwarf plants UFU MC TOM 1 (donor parent), UFU-DTOM 441111, and UFU-DTOM 19142121 (55.86, 55.10, and 55.90, respectively). Hybrids 1 and 2 showed intermediate values together with the female parent, UFU-T7A but did not differ from the cv. Santa Clara. Hybrids 3 and 4 were similar to female parents UFU-057 and UFU-T4R2#4.

The fruit shape in the hybrids was similar to that of female parents, with values below 1. The donor parent had an elongated fruit shape (1.96). The male parents and the cv. Santa Clara showed intermediate FS values; no difference was observed compared to the commercial cultivar.

The lowest number of locules was observed in the donor parent and the cv. Santa Clara (2.00 and 2.03, respectively). On the other hand, parents UFU-057 and UFU-T4R2#4 stood out regarding this parameter (5.53 and 6.70, respectively). The four hybrids showed intermediate NLs, similar to UFU-T7A, UFU-DTOM 441111, and UFU-DTOM 19142121. Control cultivar Santa Clara did not differ from hybrid 4, the donor parent, and the male parent.

The soluble solids content (BRIX) stood out positively in the donor parent (6.95). At the other extreme, the lowest value was observed in the commercial cultivar Santa Clara (4.63). The other treatments showed intermediate values for this parameter. The female parent UFU-T7A, male parent UFU-DTOM 441111, and hybrid 2 were similar to the control cultivar (Santa Clara) by Dunnett’s test.

The highest yield was obtained by hybrid 3 (106.18 t ha^−1^), standing out from the other hybrids evaluated. Dwarf plants exhibited the lowest yields. Among the normal-sized plants, the parental UFU-T4R2#4 and the cv. Santa Clara had the lowest yields and were considered similar.

The hybrids 1 to 4 exhibited the highest average fruit weights (105.57, 95.11, 111.23, and 94.35 g, respectively) along with the female parents. However, hybrids 2 and 4 did not differ from the cv. Santa Clara (Dunnett’s test, 5% probability). The lowest AFWs were observed in the dwarf plants. Among the normal-sized plants, the cv. Santa Clara had the lowest average fruit weight (72.54 g).

The highest NFP was identified in the donor parent (35.21). Among the hybrids, the highest NFP value was observed in hybrid 3 (24.25). The lowest NFP was observed in male parents, cv. Santa Clara, and female parents, with the exception of UFU-T4R2#4. Moreover, these treatments were similar to the cv. Santa Clara by Dunnett’s test, except for the parental UFU-DTOM 19142121.

**Figure 2:**
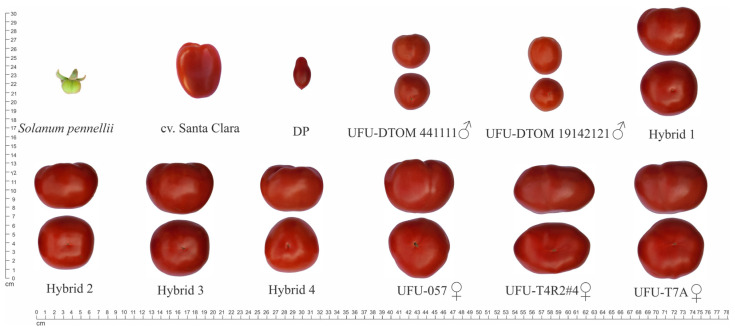
Fruit shape obtained by crossing dwarf plants with normal plants of the Salad-type and the control plants. *Solanum pennellii* (wild accession LA-716); cv. Santa Clara: commercial cultivar Santa Clara; DP: donor parent (UFU MC TOM 1); Hybrid 1: UFU-057♀ × UFU-DTOM 441111♂; Hybrid 2: UFU-T7A♀ × UFU-DTOM 441111♂; Hybrid 3: UFU-T4R2#4♀ × UFU-DTOM 441111♂; Hybrid 4: UFU-T7A♀ × UFU-DTOM 19142121♂.

It is possible to observe the agronomic potential of the dwarf male parents compared to the donor parent and the hybrids that arise from crossing dwarf plants with indeterminate growth habits against normal plants with indeterminate growth habits (Figure 2).

### 2.2. Cluster Analysis

**Figure 3:**
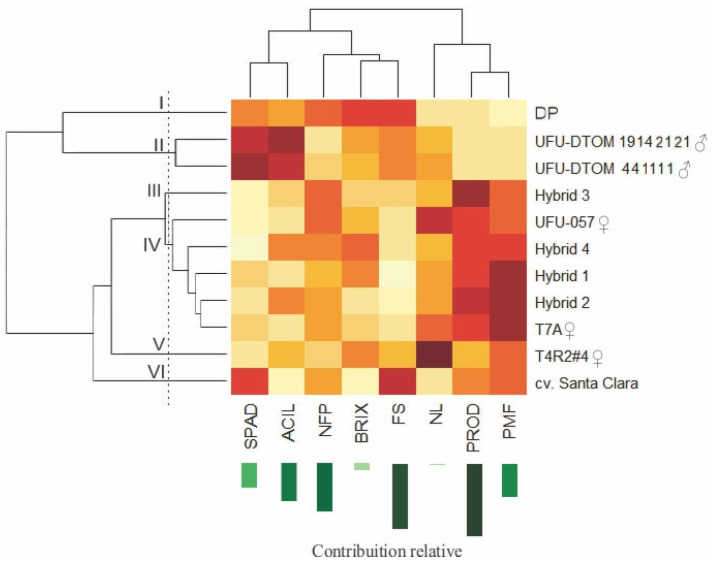
Dendrogram representing the genetic dissimilarity between tomato parents and hybrids, obtained using the UPGMA method. ACIL: acylsugar content, nmol cm^−2^ leaf area; SPAD: indirect chlorophyll content, SPAD index; FS: fruit shape; NL: number of locules; BRIX: soluble solids content, °Brix; PROD: yield, tons per hectare; PMF: Polymethoxyflavones; and NFP: number of fruits per plant. DP: donor parent (UFU MC TOM 1); Hybrid 1: UFU-057 × UFU-DTOM 441111; Hybrid 2: T7A × UFU-DTOM 441111; Hybrid 3: T4R2#4 × UFU-DTOM 441111; Hybrid 4: T7A × UFU-DTOM 19142121. Darker (more intense) colors signifying a higher response from the treatment to the variable under study.

After evaluating the agronomic performance, it was possible to analyze the genetic dissimilarity between the treatments, as shown in the dendrogram (Figure 3).

The definition of the number of groups in the dendrogram was based on the abrupt change of branches, where a visual analysis of points on the dendrogram was carried out, which enabled better discrimination of the groups [43]. The choice of this cutoff point methodology takes into account the knowledge that the researcher has regarding the evaluated germplasm. It was possible to observe the formation of six distinct groups: group I was formed by the donor parent; group II: UFU-DTOM 441111 and UFU-DTOM 19142121; group III: hybrid 3; group IV: UFU-057, hybrid 4, hybrid 1, and hybrid 2; group V: UFU-T4R2#4 and group VI by cv. Santa Clara. The dendrogram showed a cophenetic correlation coefficient (CCC) of 0.9438 with a distortion of 6.40%.

The internal dendrogram portion was visualized as a heat map, with the darker (more intense) colors signifying a higher response from the treatment to the variable under study. In general, the male parents and the donor parent had the highest ACIL and BRIX levels. The hybrids and the female parent, UFU-T7A, stood out in terms of PROD and AFW.

PROD was the trait that contributed most to genetic dissimilarity (25.20%), followed by FS (22.11%), and NFP (16.90%).

### 2.3. Kohonen Self-Organizing Map (SOM)

**Figure 4:**
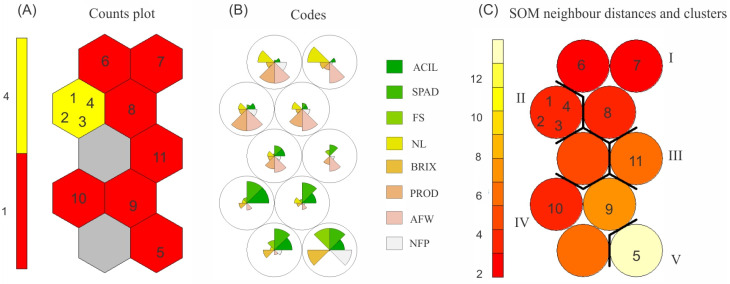
(**A**) Classification of treatments as a function of the number of neurons. (**B**) Representation of neurons and the magnitude of influence of the variables. (**C**) Nearest-neighbor distances and clustering of neurons based on the UPGMA method as a function of distance. 1: Hybrid 1 (UFU-057♀ × UFU-DTOM 441111♂); 2: Hybrid 2 (UFU-T7A♀ × UFU-DTOM 441111♂); 3: Hybrid 3 (UFU-T4R2#4♀ × UFU-DTOM 441111♂); 4: Hybrid 4 (UFU-T7A♀ × UFU-DTOM 19142121). 5. DP (Donor Parent UFU MC TOM 1); 6: Female Parent 1 (UFU-057♀); 7: Female Parent 2 (UFU-T4R2#4♀); 8: Female Parent 1 (UFU-T7A♀); 9: Male Parent 2 (UFU-DTOM 441111♂); 10: Male Parent 3 (UFU-DTOM 19142121♂); 11: cv. Santa Clara. ACIL: acylsugar content, nmol cm^−2^ leaf area; SPAD: indirect chlorophyll content, SPAD index; FS: fruit shape; NL: number of locules; BRIX: soluble solids content, °Brix; PROD: yield, tons per hectare; AFW: average fruit weight, grams; and NFP: number of fruits per plant. I, II, III, IV, and V: clusters obtained after grouping the treatments.

The analysis of the hybrids’ behavior using the SOM allowed observation of the allocation of treatments in each neuron, the influence of each agronomic trait on each neuron, the distance in neighboring neurons, and the clustering of distances based on the UPGMA method (Figure 4).

Hybrids 1, 2, 3, and 4 were allocated to the same neuron and stood out in terms of PROD and AFW, as shown in Figure 4A,B. Treatments involving dwarf plants (5, 9, and 10) stood out in terms of ACIL and SPAD. It is worth noting that the dwarf plant, corresponding to the donor parent, was allocated to the neuron farthest from the others, which could be correlated with the high BRIX and NFP content therein. The Santa Clara commercial cultivar was grouped in a neuron with no standout features, showing an intermediate behavior.

In relation to the nearest-neighbor distance (Figure 4C), neurons with similar colors indicate greater proximity, in contrast with neurons with different colors. Hybrids, female parents, and the UFU-DTOM 19,142,121 male parent showed greater proximity to one another. The donor parent showed the greatest distance from the other neurons, where it showed outstanding characteristics in the classification of this neuron (Figure 4B). The Santa Clara cultivar was placed at an intermediate distance from its neighbors.

Based on the inter-neuron distances, hierarchical clustering was carried out using the UPGMA method. The number of groups was the same as that used to form the dendrogram (five groups). Female parents were placed in group I, hybrids in group II, cv. Santa Clara in group III, male parents in group IV, and the donor parent in group V.

### 2.4. Obtaining Dwarf Plants with a Determinate Growth Habit

**Figure 5:**
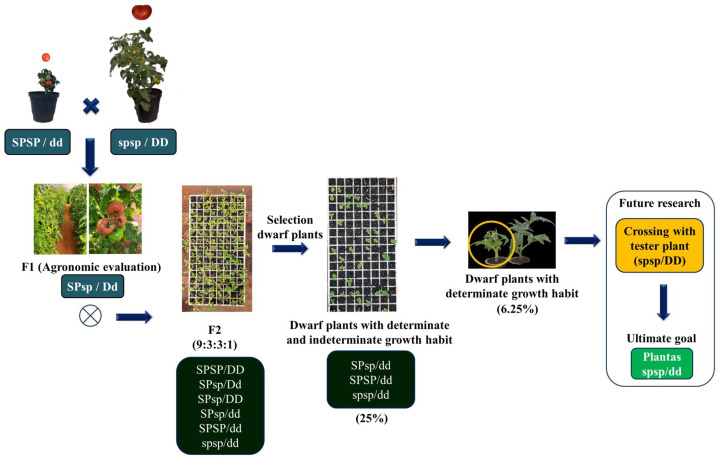
Steps to obtain dwarf plants with a determinate growth habit. SP: gene that controls the growth habit of plants; in the dominant form it conditions the indeterminate growth of plants. *dd*: dwarfism gene (dwarf), which determines the dwarf size of plants.

With the results of the previous stages in relation to agronomic performance and genetic dissimilarity, it was possible to identify the agronomic potential of the hybrids. The fruit was then harvested, and F_2_ seeds were obtained (Figure 5).

For the four self-fertilized hybrids, 450 normal-sized plants and 144 dwarf-sized plants were obtained. Fifty-five days after sowing, it was possible to visually observe within the trays the segregation into dwarf plants with determinate and indeterminate growth habits.

### 2.5. New Insights into Resistance Mechanisms from the Dwarf Plant

**Figure 6:**
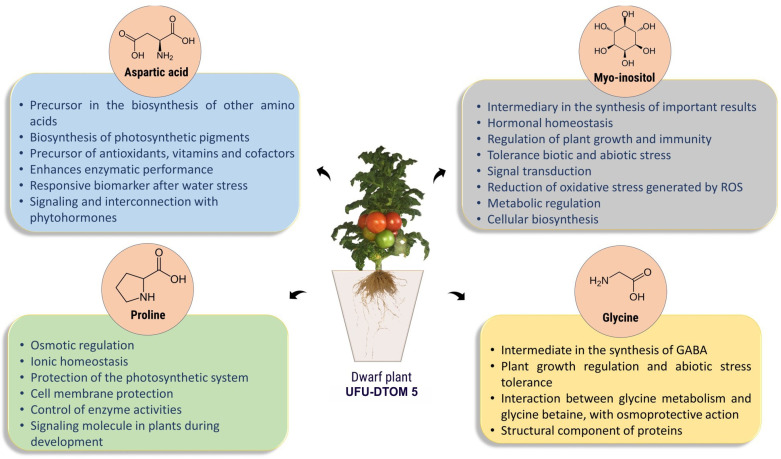
Chromatographic analysis and metabolomic profile of the main metabolites identified and their respective functions in plant metabolism.

As this is still research with incipient results on the potential use of dwarf tomato plants for pest resistance, it was necessary to investigate the presence of other compounds in the leaflets of the UFU-DTOM 5, which is a dwarf plant with a determined growth habit selected in the final stage of the project (Figure 5). By analyzing the metabolic profile of the leaves of the UFU-DTOM 5 and the commercial cultivar Santa Clara using GC-MS, a significant increase in the expression of aspartic acid, myo-inositol, proline, and glycine (significance at 5% by *t*-test) was observed. So, it was possible to observe the presence of other compounds of interest for further investigations in the search for tomato plants resistant to biotic and abiotic stresses (Figure 6).

## 3. Discussion

Different types of acylsugars can be produced by tomato plants, especially in type IV glandular trichomes [44]. The production of these secondary metabolites can promote resistance against important tomato pests [39,45]. Due to the high concentrations of these metabolites in dwarf plants, breeding them to produce hybrids is a promising approach that also offers benefits like better fruit quality [37,39,41].

It was possible to observe that the dwarf plants, donor genitor UFU MC TOM 1, and parent lines UFU-DTOM 441111 and UFU-DTOM 19142121 were superior to the others, showing the highest ACIL concentrations (Figure 1). The hybrids obtained from crossing dwarf plants with determinate growth-normal plants contained intermediate ACIL levels along with the wild access LA-716, corroborating the results obtained for mini-tomato hybrids from dwarf plants [37]. It is worth noting that all hybrids were superior to the female parents and the commercial cultivar Santa Clara, which had the lowest content of the secondary metabolite.

The identification of materials with high ACIL contents confers a greater spectrum of resistance against pests and is of great relevance in genetic improvement programs [39,41]. The use of dwarf plants to obtain hybrids with high ACIL contents could constitute a time-reducing strategy in breeding programs. The wild access LA-716 is used as a source of pest resistance due to its high acylsugar content [44,46]; however, after hybridization, there is a need to carry out backcrossing to re-establish characteristics of interest to the market [47,48,49], and the use of dwarf plants replaces this need [37].

The SPAD index is a measure that indirectly correlates the chlorophyll content in leaves [50]. Chlorophylls play important roles in photosynthesis, mainly by capturing light energy for photochemical reactions [51]. Dwarf plants showed a higher chlorophyll content, and thus possibly greater photosynthetic capacity compared to normal-sized plants.

Fruit shape is an important trait for fruit differentiation and classification. Fruits with a FS ratio of approx. 1 indicate rounded fruits [52]. In this study, the use of parents with rounded fruit made it possible to obtain hybrids with this trait, which is desirable for Salad-type tomatoes.

It was observed that the fruits of the donor parent differed from others, demonstrating that the use of backcrossing makes it possible to rescue the characteristics of the recurrent parent [41].

Fruit size can be correlated with the number of locules, where a greater NL per fruit yields larger specimens with greater mass, influencing productivity [53,54]. The donor parent had both one of the smallest NLs and the longest fruit shape. The lower number of locules results in longer-shaped fruit, resembling the shape of the Saladette tomato [55], which is undesirable for Salad-type tomatoes.

The soluble solids content is an important trait related to fruit sweetness and quality [56]. The donor parent stood out with the highest BRIX value, showing that it can be used in genetic improvement programs. The hybrids obtained from dwarf plants had intermediate BRIX levels and were superior to the Santa Clara cultivar (hybrids 1 and 2). The increase in BRIX content positively influences industrial yield [57]. This yield can be up to 20% higher for every degree of BRIX increment [58].

Breeding programs seek to obtain more productive hybrids with fruit of higher nutritional quality [37]. The use of dwarf plants as the male parent provided a hybrid with outstanding productivity (hybrid 3) compared to other hybrids and parents. Due to their architecture and size, dwarf plants are low-yielding. However, its use for hybrid production makes it possible to obtain plants that are more compact and with shorter internodes, allowing a greater number of bunches per linear meter of plants [37], resulting in yield increases.

Average fruit weight correlates with fruit shape and yield. Larger and heavier fruits have a positive impact on productivity [59]. The higher AFW, combined with larger fruit and an increase in the number of bunches per plant, leads to higher yields [60], generating larger profits for growers of this crop.

Yield is correlated with NFP [61]. Hybrid 3 showed the highest NFP and one of the highest AFW values, resulting in the highest productivity among the treatments evaluated. The opposite can also be observed in the male parents: lower AFW and NFP, influencing low productivity.

After comparing the agronomic performance, it was possible to carry out cluster analyses (Figure 3). The dendrogram satisfactorily reproduced the information contained in the dissimilarity matrix, with a CCC above 0.7 [62]. The use of dwarf plants in hybridization provided superior hybrids in terms of PROD and AFW, as observed in mini-tomato hybrids [37]. Another advantage provided by dwarf plants is their acylsugar content. This secondary metabolite is associated with greater pest resistance [39,41]. Both male parents stood out in terms of this trait, showing the potential for use in tomato breeding programs to generate resistant genotypes.

In relation to the second method of cluster analysis (Figure 4), based on artificial neural networks with the formation of a Kohonen self-organizing map, there was no coherence of grouping in relation to the dendrogram, where different groups were formed with different treatments in each one, corroborating the results observed by other authors [40,63]. Using the SOM, all hybrids were grouped in the same neuron, which showed a large distance from the neighboring neuron belonging to the Santa Clara cv., showing the dissimilarity between these treatments.

Discrepancies were also evident in relation to the donor parent, which showed the greatest distance between the hybrids and the female parents. This is due to the plant’s architecture being very different from that found in hybrids. Dwarf plants do not have the structure to support a large number of fruits of similar size and weight to normal-sized plants. However, the dwarf plants used as male parents underwent three backcrosses until the hybridization stage with the female parents, rescuing part of the characteristics of the recurrent parent, which is of normal size. This explains the distance between the treatments within the classification obtained by the neurons. Backcrossing is widely used in tomato breeding programs [38,64,65].

Hierarchical clustering methods are widely used in studies on the genetic dissimilarity of populations [66]. Despite the important information provided by these methods in breeding programs, they can have some limitations, such as difficulty in analyzing databases with a high sample number, inconsistencies in separating accessions located in more distant branches of the dendrogram, and the presence of outliers can generate clustering distortions, thus compromising the classification [67,68,69].

The use of Kohonen’s self-organizing maps is more robust and has greater processing capacity, allowing for the manipulation of large databases without limiting their dimensionality [70]. The use of artificial intelligence and SOMs has emerged as an alternative for assessing genetic dissimilarity within breeding programs [71]. Work has been carried out using SOMs to study dissimilarity using indeterminate dwarf plants [40,41,42], demonstrating the applicability of the technique in tomato genetic improvement.

After an agronomic assessment and analysis of the similarity of the treatments, F_2_ seeds were collected and sown, and after 55 days differences were observed between the dwarf plants (Figure 5). In terms of phenotypes, a 3:1 segregation was found, which corresponds to 25% dwarf-sized plants and 75% normal-sized plants. This finding supports the data found by Maciel et al. [36]. The observation of dwarf plants demonstrates the success of the initial stage, where dwarf plants were crossed with determinate growth normal plants, showing the introgression of the dwarfism gene into the hybrids.

The selected dwarf plants were kept in trays, and after 55 days, differences were observed between the plants, where some plants were smaller than others. These plants also emitted an inflorescence at the stem apex, similar to the behavior of determinate plants. There is evidence that the plants identified are determinate dwarf plants, which is innovative since there are no reports of dwarf plants with a determinate growth habit in the literature. It is suggested that the identified dwarf plants be crossed with a homozygous test plant of normal size for subsequent self-pollination and observation of the 3:1 segregation of dwarf size, confirming the identification of determinate dwarf plants (Figure 5).

These dwarf plants with a determinate growth habit could, in the future, be crossed with homozygous strains with a normal phenotype and determinate growth habit, obtaining determinate hybrids with additional advantages [8,37].

The hybrids obtained also present promising potential in relation to the metabolites found (Figure 6), as they may be associated with promoting resistance to different types of biotic and abiotic stress in plants [72]. Among the various compounds, the metabolites myo-inositol, aspartic acid, and some amino acids were identified. These compounds participate in important processes in plant metabolism, including protection of the photosynthetic apparatus, hormonal regulation, enzymatic processes, osmoregulation, and can act as intermediates in the biosynthesis of other metabolites [72,73,74]. The presence of these compounds in dwarf plants allows for greater plant resilience against biotic and abiotic stresses that affect tomato plants. Plants more adapted to saline environments, under conditions of water stress and under attack by pests, are able to complete their cycle without significant declines in important tomato characteristics [75].

The discovery of the existence of these compounds in dwarf plants allows the exploration of the potential for their use, enabling a broad spectrum of resistance. Each identified compound can be used alone or in conjunction with other compounds, with the aim of obtaining tomato plants resistant to various stresses and reducing the need to apply chemicals related to pest control.

## 4. Materials and Methods

### 4.1. Experimental Site and Description of Genotypes

**Figure 7:**
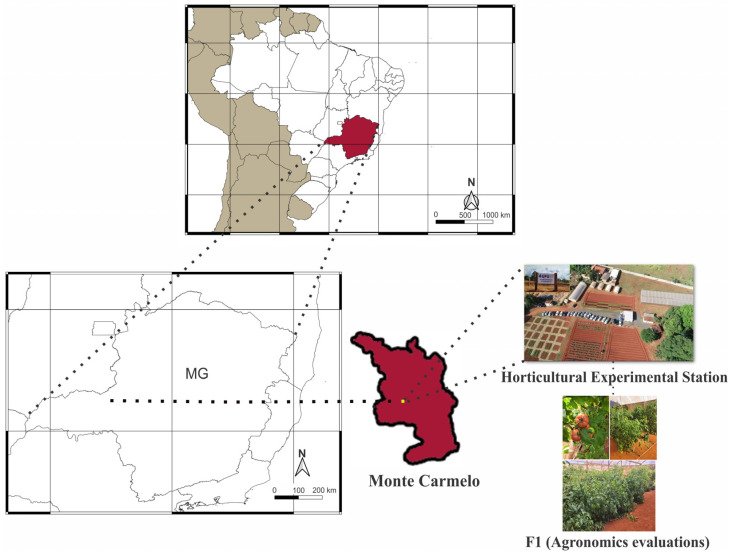
Location of the experiment, Horticultural Experimental Station (HES), UFU Monte Carmelo campus—MG.

The experiments were carried out at the Horticultural Experimental Station (HES) of the Federal University of Uberlândia (18°42′43.19″ S, 47°29′55.8″ W; altitude 873 m), Monte Carmelo campus, from 2018 to 2023 (Figure 7).

**Figure 8:**
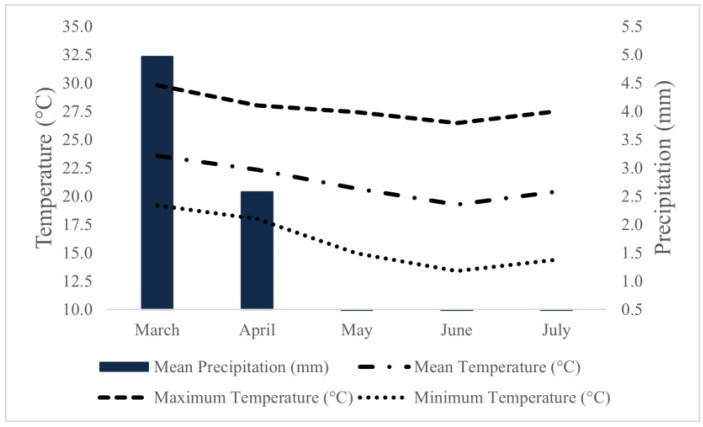
Climate chart for the period of the experiment in the city of Monte Carmelo, MG, Brazil. Source: [76].

Variations in temperature and rainfall over the course of the experiment were monitored, and the average data for the period were obtained (Figure 8).

During the experimental period, temperatures ranged between a maximum of 29.8 °C and a minimum of 13.4 °C, with an average rainfall over the period of 1.5 mm. These temperature variations were favorable to the development of tomato plants [77].

The populations of dwarf tomato plants used in this work belong to the germplasm bank of the Federal University of Uberlândia. In 2018, hybridization was carried out between UFU-57♀ and UFU MC TOM 1♂. UFU-57, used as a maternal line, is a homozygous, pre-commercial strain with normal size (dwarf gene, DD), indeterminate growth habit (SPSP), and good Salad-type agronomic characteristics (recurrent parent = UFU-57). UFU MC TOM 1, which was used as the male parent, is a dwarf (dd), indeterminate growth habit (SPSP) strain with mini-tomato type fruits (UFU MC TOM 1 = donor parent) [8,36].

**Figure 9:**
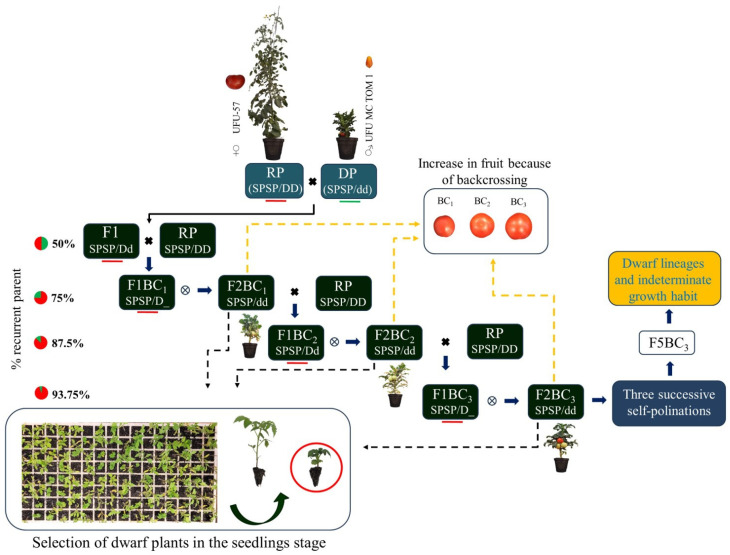
Steps to obtain the third backcrossing of dwarf plant lines with indeterminate growth habit.

After obtaining generation F_1_, the following steps were carried out between 2019 and 2022: first backcrossing (F_1_BC_1_), followed by self-pollination, yielding the F_2_BC_1_ generation (BC_1_). Dwarf plants were selected from the F_2_BC_1_ generation, and a second backcrossing (F_1_BC_2_) was carried out, followed by self-pollination, yielding the F_2_BC_2_ generation (BC_2_), from which dwarf plants were selected to carry out the third backcrossing (F_1_BC_3_), followed by self-pollination, yielding the F_2_BC_3_ generation (BC_3_). In the F_2_BC_1_, F_2_BC_2_, and F_2_BC_3_ generations, only dwarf plants and Salad-type genetic backgrounds were selected (Figure 9).

In 2022, the introgression of the self-pruning gene (*spsp*) into dwarf plants (dwarf gene, *dd*) with indeterminate growth habit (*SP*/*SP*) began (Figure 9). Three strains (UFU-057, UFU-T4R2#4, and UFU-T7A) with normal size (dwarf gene, *DD*) and determinate growth habit (gene, *spsp*) were hybridized with two dwarf strains (UFU-DTOM 441111 and UFU-DTOM 19142121; dwarf gene, *dd*) and indeterminate growth habit (gene, *SP*/*SP*), yielding four hybrids. The experiment also used donor genotype UFU MC TOM1 [36], commercial cultivar Santa Clara as a control, and wild access *Solanum pennellii* (LA-716) to compare the acylsugar content.

All treatments were sown in 128-cell polystyrene trays filled with commercial, coconut-fiber-based substrates in March 2023. The plants were grown in an arched greenhouse (7 × 21 m) with a ceiling height of 4 m, side curtains of anti-aphid mesh, and a transparent polyethylene cover to protect against UV radiation. Thirty days after sowing, the plants were transplanted into the ground in a semi-detached, arched-roof greenhouse (14 × 48 m), with a ceiling height of 4 m, side curtains made of anti-aphid mesh, and a transparent 200-micron polyethylene cover to protect against UV radiation. The experiment employed a randomized block design with 12 treatments and three replicates, representing 36 plots with seven plants each, for a total of 252 plants in the experiment. Cultivation was carried out as recommended for tomato cultivation [6].

### 4.2. Agronomic Evaluations

Acylsugar content (ACIL) (nmol cm^−2^ of leaf area): obtained at 75 days after sowing (DAS) by taking a sample, in duplicate, made up of eight leaf discs, equivalent to 4.2 cm^2^. The discs were collected from the middle third of the plants and inserted into test tubes for extraction and quantification according to the methodology adapted from Maciel and Silva [78]. Wild access *S. pennellii* (LA-716) was used to compare data for this agronomic trait.

SPAD index (SPAD): obtained using the Minolta SPAD-502 CFL1030 chlorophyll meter and based on the average of the readings on the leaflets of the middle third of the plants, producing the indirect chlorophyll content [50].

Fruit shape (FS): obtained from the ratio between transverse and longitudinal diameter; values close to one indicate round fruit.

Number of locules (NL): observed after the horizontal cut in the central region of the fruit.

Total fruit soluble solids content (°Brix): analyzed at the point of full fruit ripeness using a portable digital refractometer (Atago PAL-1 3810).

Productivity (kg ha^−1^) (PROD): obtained from the number of plants per hectare at a spacing of 0.4 m × 1.5 m, multiplied by the yield of each plant.

Average number of fruits per plant (NFP): obtained from the ratio between the total number of fruits and the number of plants per plot.

Average fruit weight (AFW): obtained from the ratio between fruit mass and fruit number in each plot.

### 4.3. Chromatographic Analysis and Metabolomic Profile

Leaflet samples (n = 6) of the UFU-DTOM 5 genotype and commercial cultivar Santa Clara using were collected from the middle portion of the plant and crushed with liquid nitrogen using a mortar and pestle until a fine powder was obtained. From this powder, 100 mg was transferred to an Eppendorf microtube and mixed with 2 mL of an extracting solution composed of methanol (MeOH), chloroform (CHCl_3_), and ultrapure water (in a 3:1:1 ratio), containing 50 µL/mL of Adonitol Purex as an internal standard. The samples were shaken for 5 s using a vortex shaker and allowed to rest for 1 min. Subsequently, 1 mL of the supernatant was transferred to a new microtube and mixed with 300 µL of hexane (P.A.). The samples were left for 3 min, and then 500 µL of the middle portions were collected and placed in microtubes for drying in a concentrator at 40 °C for 24 h. To the dried samples, 50 µL of methoxyamine hydrochloride diluted in pyridine (20 mg/mL) was added at 37 °C. After 2 h, 50 µL of BSTFA (Bis(trimethylsilyl) trifluoroacetamide) was added, and the samples were kept at 37 °C for 30 min. The derivatized aliquots were transferred to 2 mL vials with 200 µL volume-reducing inserts for chromatographic analysis.

The analysis was performed by gas chromatography-mass spectrometry (GCMS-QP2010, Shimadzu, Kyoto, Japan), using a DB-5MS capillary column (30 m × 250 μm internal diameter). The sample injection temperature used was 250 °C. Chromatographic separation was carried out with an initial column temperature of 80 °C, which was maintained for 2 min, then increased at a rate of 5 °C/min until 250 °C. This final temperature was maintained for 5 min, with a constant flow of helium gas at 1.0 mL/min. The injection volume was 1 μL with a 10:1 split ratio. Mass spectra were scanned in the range of 40 to 650 *m*/*z* in full scan mode, at 5 scans per second. A 3 min solvent cutoff was applied, considering the retention time of pyridine used in the derivatization step. The interface and ion source temperatures used were 280 °C. The detector voltage was set at 1.2 kV, and electron impact (EI) ionization mode was selected for metabolite ionization at 70 eV. A standard of alkanes (C9–C30) was used for quality control and retention index calculations. Compound identification was conducted using the NIST Mass Spectral Library 2017, focusing on compounds with hits exceeding 85% similarity and matching *m*/*z* values.

### 4.4. Statistical Analysis

After checking assumptions using the homogeneity of variance analysis (Levene’s test), normality (ONeil–Mathews test), and additivity (test of Tukey additivity), values were tabulated. The data were submitted to analysis of variance using the F-test (*p* < 0.05). Means were compared using the Scott–Knott test (*p* < 0.05). Dunnett’s test (*p* < 0.05) was used to compare the performance of the treatments in relation to the cv. Santa Clara.

Multivariate analysis of genetic dissimilarity between the genotypes was subsequently carried out using the generalized Mahalanobis distance ($D_{\mathrm{ii}’}^{2}$). A dendrogram obtained using the unweighted pair-group method using arithmetic averages (UPGMA) hierarchical method was used to represent genetic divergence. The validation of clustering using the UPGMA method was determined by the cophenetic correlation coefficient (CCC) calculated using the Mantel test [79]. The relative contribution of the quantitative characters was calculated according to Singh’s criteria [80]. All obtained data was analyzed using Genes software v. 1990.2021.131 [81].

Moreover, an unsupervised data classification was carried out using a Kohonen self-organizing map (SOM). There is no specific methodology for determining the parameters needed to generate the map [68,82]. Different neuron arrays were tested—2 × 2, 3 × 3, 4 × 2, and 2 × 5—with the latter arrangement being chosen for classification. Four hundred interactions were used for the learning process, with a radius equal to one and a hexagonal topology. After obtaining the treatments in each neuron and their distance from neighboring neurons, they were hierarchically grouped using the UPGMA method.

The analyses were carried out using GENES software v. 1990.2021.131, integrated within Matlab v. R2016a [81] and R v. 4.2.1 software packages [83]. The Kohonen package [84] was used to generate the SOM in the R software.

After evaluating the agronomic performance of the hybrids, fruit was collected for seed extraction, and the F_2_ generation was subsequently sown. Thirty days after sowing, a visual count of dwarf plants and normal plants was carried out to obtain the segregation ratio [36]; dwarf plants were selected to identify those with a determinate growth habit.

## 5. Conclusions

Hybrids obtained from dwarf male parents showed satisfactory agronomic performance, with hybrid 3 standing out as having the highest yield.

After self-pollination of the F1 generation, it was possible to obtain dwarf plants with a determinate growth habit, confirming the introgression of the self-pruning gene.

It was possible to observe visual differences between determinate and indeterminate dwarf plants when the seedlings were still developing in the trays.

The dwarf tomato plant has great potential for exploration and use in tomato genetic improvement programs with an emphasis on biotic and abiotic stresses.

## Data Availability

Data are contained within the article.
